# Epigenetic silencing of TMEM176A activates ERK signaling in human hepatocellular carcinoma

**DOI:** 10.1186/s13148-018-0570-4

**Published:** 2018-11-06

**Authors:** Hongxia Li, Meiying Zhang, Enqiang Linghu, Fuyou Zhou, James G. Herman, Liming Hu, Mingzhou Guo

**Affiliations:** 1 College of Life Science and Bioengineering, Beijing University of Technology, Beijing, 100124 China; 2 Department of Gastroenterology and Hepatology, Chinese PLA General Hospital, #28 Fuxing Road, Beijing, 100853 China; 3 Department of Thoracic Surgery, Anyang Tumor Hospital, Anyang, 455000 China; 4 The Hillman Cancer Center, University of Pittsburgh Cancer Institute, 5117 Centre Avenue, Suite 2.18/Research, Pittsburgh, PA 15213 USA

**Keywords:** TMEM176A, DNA methylation, HCC, SAR1A, ERK1/2

## Abstract

**Background:**

The role of TMEM176A in human hepatocellular carcinoma (HCC) is unknown. This study explored the epigenetic regulation and function of TMEM176A in human HCC.

**Materials and methods:**

Twelve HCC cell lines and 126 cases of primary cancer were analyzed. Methylation-specific PCR, immunohistochemistry, flow cytometry, and xenograft mouse models were employed.

**Results:**

TMEM176A was highly expressed in SNU387, SNU182, Huh1, and SNU475 cells; reduced expression was observed in HepG2 and PLC/PRF/5 cells; and no expression was found in SNU449, HBXF344, SMMC7721, Huh7, and LM3 cells. Unmethylation of the TMEM176A promoter was detected in SNU387, SNU182, Huh1, and SNU475 cells; partial methylation was observed in HepG2 and PLC/PRF/5 cells; and complete methylation was found in SNU449, HBXF344, SMMC7721, Huh7, and LM3 cells. Upon treatment with 5-Aza-2-deoxycytidine, re-expression of TMEM176A was detected in SNU449, HBXF344, SMMC7721, Huh7, and LM3 cells; increased expression of TMEM176A was observed in HepG2 and PLC/PRF/5 cells; and no expression changes were found in SNU387, SNU182, Huh1, and SNU475 cells. The TMEM176A promoter region was methylated in 75.4% (95/126) of primary human HCC. Reduced expression of TMEM176A was associated with promoter region methylation (*P* < 0.05). No association was found between TMEM176A promoter methylation and age, gender, HBV infection, liver cirrhosis, tumor size, lymph node metastasis, vessel cancerous embolus, number of lesions, and TNM stage (all *P* > 0.05). These results demonstrated that the expression of TMEM176A is regulated by promoter region methylation. Methylation of the TMEM176A promoter was significantly associated with tumor cell differentiation (*P* < 0.05) and was an independent prognostic factor for poor 3-year overall survival (OS, *P* < 0.05). TMEM176A expression induced cell apoptosis; inhibited cell proliferation, migration, and invasion; suppressed human HCC cell xenograft growth in mice; and inhibited ERK signaling in HCC cells.

**Conclusion:**

The promoter region of TMEM176A is frequently methylated in human HCC, and the expression of TMEM176A is regulated by promoter region methylation. Methylation of the TMEM176A promoter may serve as a diagnostic and prognostic marker in HCC. TMEM176A suppresses HCC growth by inhibiting the ERK signaling pathway.

## Introduction

Hepatocellular carcinoma (HCC) is one of the most common cancers and the third leading cause of cancer-related death worldwide [1, 2]. HCC is related to hepatitis B and hepatitis C virus infection, and in China, it is mainly related to hepatitis B infection [3]. Despite the improvement in surgical techniques, the prognosis of HCC remains poor due to the lack of effective prediction and prognostic markers. Aberrant genetic and epigenetic changes are regarded as important mechanisms of human cancers, including HCC [4–9].

The membrane-spanning 4A (MS4A) gene family includes 24 distant human and mouse genes. With the exception of MS4A6E, which contains two transmembrane domains, all family members have at least four transmembrane domains and N- and C-terminal cytoplasmic domains encoded by distinct exons. All MS4A genes are clustered on chromosome 11q in humans in a region with linkage to allergy [10]. Two MS4A-related genes, TMEM176A and TMEM176B, are located on chromosome 7. TMEM176B is broadly expressed and has been shown to be upregulated in antigen-presenting cells in a rat model of allograft tolerance [11]. Human TMEM176A was first identified from a screen of tumor-associated antigens in HCC [12]. Human TMEM176B was first discovered in human lung fibroblasts [13] and was found to be associated with human small cell lung cancer [14]. In a recent study, TMEM176A and TMEM176B transcripts were undetected or detected at only trace levels in most samples of normal human blood or tonsillar B cells, and tissue expression was broad for both TMEM176 genes, suggesting that they have generalized rather than cell type-specific functions [15]. TMEM176A is located in human chromosome 7q36.1, a region where there is a frequent loss of heterozygosity in human cancer [16, 17]. In our previous studies, the TMEM176A promoter was frequently methylated in human colorectal and esophageal cancers and served as a tumor suppressor in these cancers [18, 19].

In this study, we investigated the epigenetic regulation of TMEM176A and further explored the role of TMEM176A in HCC. We found that TMEM176A is frequently methylated in human HCC. Methylation of TMEM176A is associated with tumor cell differentiation and poor 3-year overall survival (OS). The expression of TMEM176A is regulated by promoter region methylation. TMEM176A suppressed human HCC cell growth both in vitro and in vivo. TMEM176A suppresses HCC cell growth by inhibiting ERK signaling through interacting with SAR1A in HCC cells. Silencing of TMEM176A by promoter region hypermethylation may activate the ERK signaling pathway and promote tumorigenesis in human HCC.

## Materials and methods

### Human tissue samples and cell lines

Primary HCC samples (126) were collected from the Chinese PLA General Hospital. The median age of the cancer patients was 55 years old (range from 29 to 79). Fifteen cases of normal liver tissue were collected from the Chinese PLA General Hospital. Among 126 cancer samples, only 41 cases were available for paraffin samples with matched cancer and adjacent tissue. All samples were collected following the guidelines approved by the Institutional Review Board of the Chinese PLA General Hospital with written informed consent from patients. Twelve HCC cell lines (SNU182, SNU449, HBXF344, SMMC7721, Huh7, HepG2, LM3, PLC/PRF/5, BEL7405, SNU387, SNU475, and Huh1) were previously established from primary HCC [20] and grown in RPMI-1640 (Invitrogen, Carlsbad, CA, USA) supplemented with 10% fetal bovine serum (Hyclone, Logan, UT) and 1% penicillin*/*streptomycin solution (Sigma, St. Louis, MO).

### 5-Aza-2-deoxycytidine and SCH772984 treatment

For methylation regulation analysis, HCC cell lines were split to low density (30% confluence) 12 h before treatment. Cells were treated with 5-Aza-2′-deoxycytidine (DAC, Sigma, St. Louis, MO, USA) at a concentration of 2 μM in the growth medium, which was exchanged every 24 h for a total of 96 h and cultured at 37 °C in a 5% CO2 incubator. At the end of the treatment period, cells were prepared for extraction of total RNA. To verify the role of TMEM176A in ERK signaling, SCH772984, an ERK inhibitor, was added to TMEM176A knocking down SNU387 and SNU475 cells at 1 μm and 4 μm for 24 h (MedChemExpress, Monmouth Junction, USA) [21].

### RNA isolation and semi-quantitative RT-PCR

Total RNA was extracted using Trizol Reagent (Life Technologies, Carlsbad, CA, USA). Agarose gel electrophoresis and spectrophotometric analysis were used to detect RNA quality and quantity. First-strand cDNA was synthesized according to the manufacturer’s instructions (Invitrogen, Carlsbad, CA). A total of 5 μg RNA was used to synthesize the first-strand cDNA. The reaction mixture was diluted to 100 μl with water, and then 2 μl of diluted cDNA was used for 25 μl PCR reaction. The PCR primer sequences for TMEM176A were as follows: 5′-GGGAACAG CCG ACA G TGAT-3′ (F) and 5′-GCC AGC GTT AGCAGAGTCCT-3′ (R). PCR cycle conditions were as follows: 95 °C 5 min, 1 cycle; 95 °C 30 s, 60 °C 30 s, and 72 °C 30 s, 32 cycles; and 72 °C 5 min, 1 cycle. PCR product size is 369 bp. GAPDH was amplified for 25 cycles as an internal control. The GAPDH primer sequences were as follows: 5′-GACCAC AGT CCA TGC CAT CAC-3′ (F) and 5′-GTC CACCAC CCT GTT GCT GTA-3′ (R). PCR cycle conditions were as follows: 95 °C 5 min, 1 cycle; 95 °C 30 s, 63 °C 30 s, and 72 °C 30 s, 25 cycles; and 72 °C 5 min, cycle. PCR product size is 448 bp. The amplified PCR products were examined by 2% agarose gels.

### DNA extraction, bisulfite modification, and methylation-specific PCR

Genomic DNA from HCC cell lines and HCC tissue samples were prepared using the proteinase K method. Normal lymphocyte DNA was prepared from healthy donor blood lymphocytes by proteinase K method [22]. Normal lymphocyte DNA (NL) was used as a control for unmethylation and in vitro-methylated DNA (IVD) was used as a methylation control. IVD was prepared using SssI methylase (New England Biolabs, Ipswich, MA, USA) following the manufacturer’s instructions. Methylation-specific PCR (MSP) primers were designed according to genomic sequences inside the CpG islands in the TMEM176A gene promoter region.

MSP primers for TMEM176A were designed − 364 to − 203 bp upstream of the transcription start site (TSS) and synthesized to detect methylated (M) and unmethylated (U) alleles. The detected region has been previously reported to be hypermethylated and associated with low expression [19]. MSP primers for TMEM176A were as follows: 5′-GTTTC GTTTA GGTT GCGC GG TTT TTC-3′ (MF) and 5′-CCAAA ACCGACGTA CAAATA TACG CG-3′ (MR); 5′-TGGTTTTGTTTAGGTTGTGTGGTTTTTT-3′ (UF) and 5′-CAA CCA AAA CCAACAT ACAAAT ATACA CA-3′ (UR).

PCR cycle conditions were as follows: 95 °C 5 min, 1 cycle; 95 °C 30 s, 60 °C 30 s, and 72 °C 30 s, 35 cycles; and 72 °C 5 min, 1 cycle.

Bisulfite sequencing (BSSQ) primers encompassed a 231-bp region upstream of the TMEM176A transcription start site (− 388 to − 157 bp) and included the region analyzed by MSP. BSSQ primers were designed as follows: 5′-GAG ACG GTA GAT GTA CGG GT-3′ (F) and 5′-AAC RAA CRA CCC TAA AAA AAC CC-3′ (R). PCR cycle conditions were as follows: 95 °C 5 min, 1 cycle; 95 °C 30 s, 55 °C 30 s, and 72 °C 30 s, 35 cycles; and 72 °C 5 min, 1 cycle.

### Immunohistochemistry

Immunohistochemistry (IHC) was performed in primary HCC samples and matched adjacent tissue samples. TMEM176A antibody was diluted to 1:50 (Cat: HPA008770, Sigma, St. Louis, MO, USA). The expression of SAR1A was detected in LM3 cell xenografts. SAR1A antibody was diluted to 1:200 (Protein Tech Group, Chicago, IL, USA). The procedure was performed as described previously [6]. The staining intensity and extent of the staining area were scored using the German semi-quantitative scoring systems as previously described [6, 9, 16, 23]. Staining intensity of the membrane and/or cytoplasm was characterized as follows: no staining = 0, weak staining = 1, moderate staining = 2, and strong staining = 3; the extent of staining was defined as follows: 0% = 0, 1–24% = 1, 25–49% = 2, 50–74% = 3, and 75–100% = 4. The final immune-reactive score (0–12) was determined by multiplying the intensity score by the extent of staining score.

### Construction of lentiviral TMEM176A expression vectors and selection of stable expression cells

The human full-length TMEM176A cDNA (NM-018487.2) was cloned into the pLenti6 vector. The primers were as follows: 5′-CTTAGGATCCGCCACCATGGGAACAGCCGAC-3′ (F) and 5′-ACTTAGTCGACCTAGATTCCACTCACTTCC-3′ (R). The HEK-293T cell line was maintained in DMEM (Invitrogen, CA, USA) supplemented with 10% fetal bovine serum. TMEM176A expressing lentiviral vector was transfected into HEK-293T cells (5.5 × 10^6^ per 100 mm dish) using Lipofectamine 3000 Reagent (Invitrogen, Carlsbad, CA, USA) at a ratio of 1:3 (DNA mass to Lipo mass). Viral supernatant was collected and filtered after 48 h. LM3 and SNU449 cells were then infected with a viral supernatant. Cells stably expressing TMEM176A were selected with Blasticidin (Life Technologies, Carlsbad, CA, USA) at concentrations of 2.0 μg/ml for 2 weeks.

### RNA interference assay

Two sets of targeting siRNA for TMEM176A and one set of RNAi negative control duplex sequence are as follows: SiTMEM176A1 duplex (sense: 5′-GGCUACUCUUAUUACAACATT-3′; antisense: UGUUGUAAUAAGAGUAGCCTT-3′), SiTMEM176A2 duplex (sense: 5′-CUGUACUGCUGGAGAAUGUTT-3′; antisense: 5′-ACAUUCUCCAGCAGUACAGTT-3′) and SiTMEM176A negative control duplex (SiTMEM176ANC, sense: 5′-ACAUUCUCCAGC AGUACAGTT-3′; antisense: 5′-ACGUGACACGUUCGGAGAATT-3′). SiTMEM176A2 was found more effective than SiTMEM176A1, and SiTMEM176A2 was applied to further study (GenePharma Co. Shanghai, China).

Three sets of targeting siRNA for SAR1A1 and one set of RNAi negative control duplex sequence are as follows: SiSAR1A1 duplex (sense: 5′-CCUAGGACUGUACAAGAAATT; antisense: 5′-UUUCUUGUACAGUCCUAGGTT-3′), SiSAR1A2 duplex (sense: 5′-CCAACACUACAUCCGACAUTT-3′, antisense: 5′-AUGUCGGAUGUAGUGUUGGTT-3′), SiSAR1A3 duplex (sense:5′-CCAAUGUGCCAAUCCUUAUTT-3′, antisense: 5′-AUAAGGAUUGGCACAUUGGTT-3′), and SiSAR1A negative control duplex (SiSAR1ANC, sense: 5′-ACAUUCUCCAGC AGUACAGTT-3′; antisense: 5′-ACGUGACACGUUCGGAGAATT-3′). SiSAR1A1 was found more effective than SiSAR1A2 and SiSAR1A3, and SiSAR1A1 was applied to further study (GenePharma Co. Shanghai, China).

### Cell viability detection

LM3 and SNU449 cells were seeded into 96-well plates before and after the re-expression of TMEM176A at 1 × 10^3^ cells per well. SNU387 and SNU475 cells were plated into 96-well plates before and after the knockdown of TMEM176A at a density of 2 × 10^3^ cells per well. The cell viability was measured by MTT(3-(4,5)-dimethylthiahiazo (-z-y1)-3,5-di-phenytetrazoliumromide) assay at 0 h, 24 h, 48 h, 72 h, and 96 h (KeyGENBiotech, Nanjing, China). Absorbance was measured on a microplate reader (Thermo Multiskan MK3, MA, USA) at a wavelength of 490 nm. Each experiment was repeated three times.

### Colony formation assay

TMEM176A stably re-expressed and unexpressed LM3, and SNU449 cells were plated onto 6-well plates at a density of 200 cells per well. SNU387 and SNU475 cells before and after the knockdown of TMEM176A were seeded in 6-well plates at a density of 200 cells per well. After 2 weeks, cells were fixed with 75% ethanol for 30 min. Colonies were then stained with 0.5% crystal violet solution and counted. The experiment was performed in triplicate.

### Flow cytometry

To increase the sensitivity of apoptosis detection, TMEM176A stably unexpressed and re-expressed LM3 and SNU449 cells were treated with doxorubicin at 0.8 μg/ml and 0.6 μg/ml for 24 h, respectively [24]. Apoptosis was also analyzed in SNU387 and SNU475 cells with or without knockdown of TMEMA176. The cells were prepared using the FITC Annexin V Apoptosis Detection Kit I (BD Biosciences, Franklin Lakes, NJ, USA) following the manufacturer’s instructions and then sorted by FACS Calibur (BD Biosciences, Franklin Lakes, NJ, USA). Each experiment was repeated three times.

### Transwell assay

#### Migration

2 × 10^4^ TMEM176A unexpressed and re-expressed LM3 and 2 × 10^5^ SNU449 cells were suspended in 200 μl serum-free RPMI 1640 media and added to the upper chamber of an 8.0-μm pore size transwell apparatus (COSTAR Transwell Corning Incorporated, Tewksbury, MA, USA). Cells that migrated to the lower surface of the membrane were stained with crystal violet and counted in three independent high-power fields (× 100) after incubation for 16 h (LM3 cells) or 48 h (SNU449). SNU387 and SNU475 cells (1 × 10^4^) before and after knockdown of TMEM176A were added to the upper chamber of an 8.0-μm pore size transwell apparatus. Cells were migrated to the lower surface of the membrane after incubating for 12 h (SNU387) and 10 h (SNU475). Each experiment was repeated three times.

#### Invasion

The top chamber was coated with a layer of extracellular matrix. LM3 cells (8 × 10^4^) and SNU449 cells (2 × 10^5^) were seeded to the upper chamber of a transwell apparatus coated with Matrigel (BD Biosciences, CA, USA) and incubated for 36 h (LM3) and 56 h (SNU449). SNU387 and SNU475 cells (5 × 10^4^) were added to the upper chamber of a transwell apparatus coated with Matrigel before and after knockdown of TMEM176A. After 24 h incubation, cells that invaded the lower membrane surface were stained with crystal violet and counted in three independent high-power fields (× 100). Each experiment was repeated three times.

### Western blot

Cells were collected 48 h after transfection, and cell lysates were prepared using ice-cold Tris buffer (20 mmol/l Tris; pH 7.5) containing 137 mmol/l NaCl, 2 mmol/l EDTA, 1% Triton X, 10% glycerol, 50 mmol/l NaF, 1 mmol/l DTT, PMSF, and a protein phosphatases inhibitor (Applygen Tech., Beijing, China). For extracellular signal-regulated kinase (ERK) signaling analysis, cells were starved with serum-free medium for 24 h after transfection. These cells were then stimulated with a medium containing 10% serum for 45 min before collection. Western blot was performed as described previously [6]. Primary antibodies were as follows: TMEM176A (Sigma, St. Louis, MO), cleaved caspase-3 (Cell Signaling Technology, Danfoss, MA, USA), MMP2 (Bioworld Tech., MN, USA), MMP9 (Bioworld Tech., MN, USA), ERK1/2 (Protein Tech Group, Chicago, IL, USA), p-ERK1/2 (Cell Signaling Technology, Danfoss, MA, USA), SAR1A (Protein Tech Group, Chicago, IL, USA), and β-actin (Beyotime Biotech, Nanjing, China).

### Immunoprecipitation

Immunoprecipitation (IP) was performed by using antibodies against Flag (Protein Tech Group, Chicago, IL, USA) and protein A/G Agarose (Thermo Scientific, Carlsbad, CA, USA). LM3 cells were transiently transfected with Flag-tagged TMEM176A or empty vector using Lipofectamine 3000 Reagent (Invitrogen, Carlsbad, CA, USA) at a ratio of 1:2 (DNA mass to Lipo mass) in a 100-mm-diameter dish. LM3 cells were lysed in IP buffer (Thermo Scientific, Carlsbad, CA, USA). Cell lysates were incubated with antibodies for 12 h at 4 °C and then with protein A agarose beads for 4 h at 4 °C. Beads were washed with IP lysis buffer three times, and bound proteins were eluted with × 5 loading buffer and analyzed by Western blot with indicated antibodies. A Rabbit IgG antibody (Biodragon, Beijing, China) was used as a negative control.

### HCC cell xenograft mouse model

LM3 cell lines stably transfected with plenti6 vector or plenti6-TMEM176A vector (6 × 10^6^ cells diluted in phosphate-buffered saline) were injected subcutaneously into the dorsal left side of 4-week-old female Balb/c nude mice. Each group included six mice. Tumor volume was measured every 4 days. Tumor volume was calculated according to the formula: *V* = *L* × *W*^2^/2, in which *V* represents volume (mm^3^), *L* represents the biggest diameter (mm), and *W* represents the smallest diameter (mm). Mice were sacrificed on the 24th day after inoculation, and tumors were weighed. All procedures were approved by the Animal Ethics Committee of the Chinese PLA General Hospital.

### Data analysis

RNA-Seq data for TMEM176A gene expression in the dataset of HCC and normal tissues were downloaded from The Cancer Genome Atlas (TCGA) (http://xena.ucsc.edu/, 01/26/2018). Statistical analysis was performed using SPSS 17.0 software (SPSS, Chicago, IL). Chi-square or Fisher’s exact tests were used to evaluate the relationship between methylation status and clinicopathological characteristics. The two-tailed independent samples *t* test was applied to determine the statistical significance of the differences between the two experimental groups. Survival rates were calculated by the Kaplan-Meier method, and differences in survival curves were evaluated using the log-rank test. Cox proportional hazards models were fit to determine independent associations of TMEM176A methylation with 3-year OS. Two-sided tests were used to determine the significance, and *P* < 0.05 was considered statistically significant.

## Results

### TMEM176A is silenced by promoter region hypermethylation in HCC cells

**Fig. 1:**
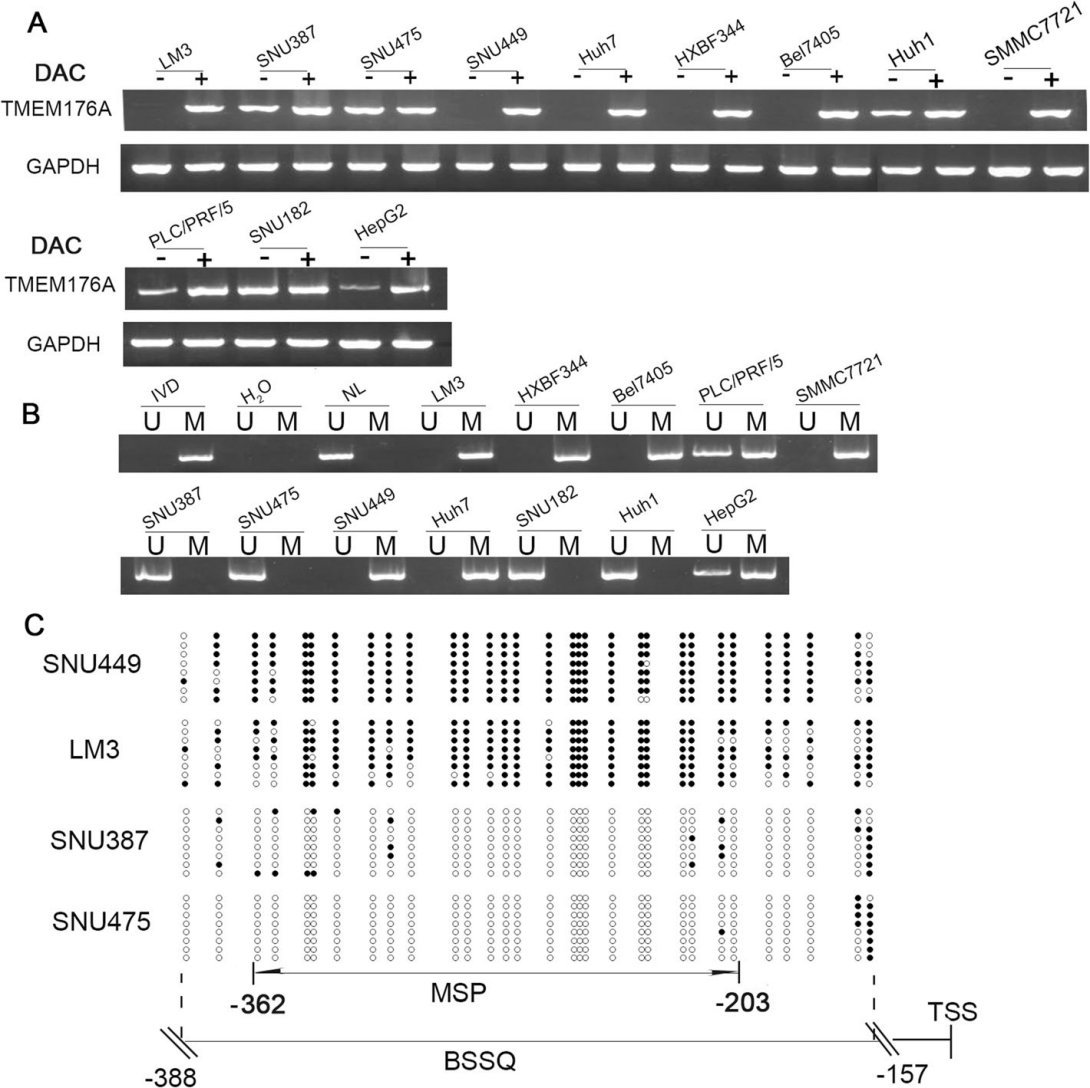
TMEM176A expression and methylation status in human HCC cells. **a** Semi-quantitative RT-PCR shows TMEM176A expression levels in HCC cell lines. SNU182, SNU449, HBXF344, SMMC7721, Huh7, HepG2, LM3, PLC/PRF/5, BEL7405, SNU387, SNU475, and Huh1 are HCC cells. DAC: 5-Aza-2′-deoxycytidine; GAPDH: internal control; (-): absence of DAC; (+): presence of DAC. **b** MSP results of TMEM176A in HCC cell lines. U: unmethylated alleles; M: methylated alleles; IVD: in vitro methylated DNA, serves as methylation control; NL: normal peripheral lymphocytes DNA, serves as unmethylated control; H_2_O: double distilled water. **c** BSSQ results of TMEM176A in LM3, SNU449, SNU387, and SNU475 cells. Double-headed arrow, MSP PCR product size was 159 bp and bisulfite sequencing focused on a 231-bp region of the CpG island (from − 388 to − 157) around the TMEM176A transcription start site. Filled circles: methylated CpG sites, open circles: unmethylated CpG sites. TSS: transcription start site

The expression of TMEM176A was examined in human HCC cells by semi-quantitative RT-PCR. TMEM176A was highly expressed in SNU387, SNU182, Huh1, and SNU475 cells; reduced expression was observed in HepG2 and PLC/PRF/5 cells; and no expression was found in SNU449, HBXF344, SMMC7721, Huh7, and LM3 cells (Fig. 1a). Promoter region methylation status was examined by methylation-specific PCR (MSP). Unmethylation was detected in SNU387, SNU182, Huh1, and SNU475 cells; partial methylation was observed in HepG2 and PLC/PRF/5 cells; and complete methylation was found in SNU449, HBXF344, SMMC7721, Huh7, and LM3 cells (Fig. 1b). These results demonstrate that the loss of/reduced expression of TMEM176A was correlated with promoter region methylation.

To further validate that the expression of TMEM176A was regulated by the promoter region methylation, HCC cells were treated with 5-Aza-2-deoxycytidine. Upon treatment with 5-Aza-2-deoxycytidine, re-expression of TMEM176A was found in SNU449, HBXF344, SMMC7721, Huh7, and LM3 cells; increased expression of TMEM176A was observed in HepG2 and PLC/PRF/5 cells; and no expression changes were found in SNU387, SNU182, Huh1, and SNU475 cells before and after treatment (Fig. 1a). These results suggest that the expression of TMEM176A is regulated by promoter region methylation in HCC cells. To further validate the efficiency of MSP primers and explore the methylation density in HCC, sodium bisulfite sequence (BSSQ) was performed in SNU449, LM3, SNU387, and SNU475 cells. Dense methylation was observed in the promoter region of TMEM176A in SNU449 and LM3 cells, and unmethylation was detected in SNU387 and SNU475 (Fig. 1c).

### TMEM176A is frequently methylated in human primary HCC, and methylation of TMEM176A is associated with poor 3-year overall survival

**Fig. 2:**
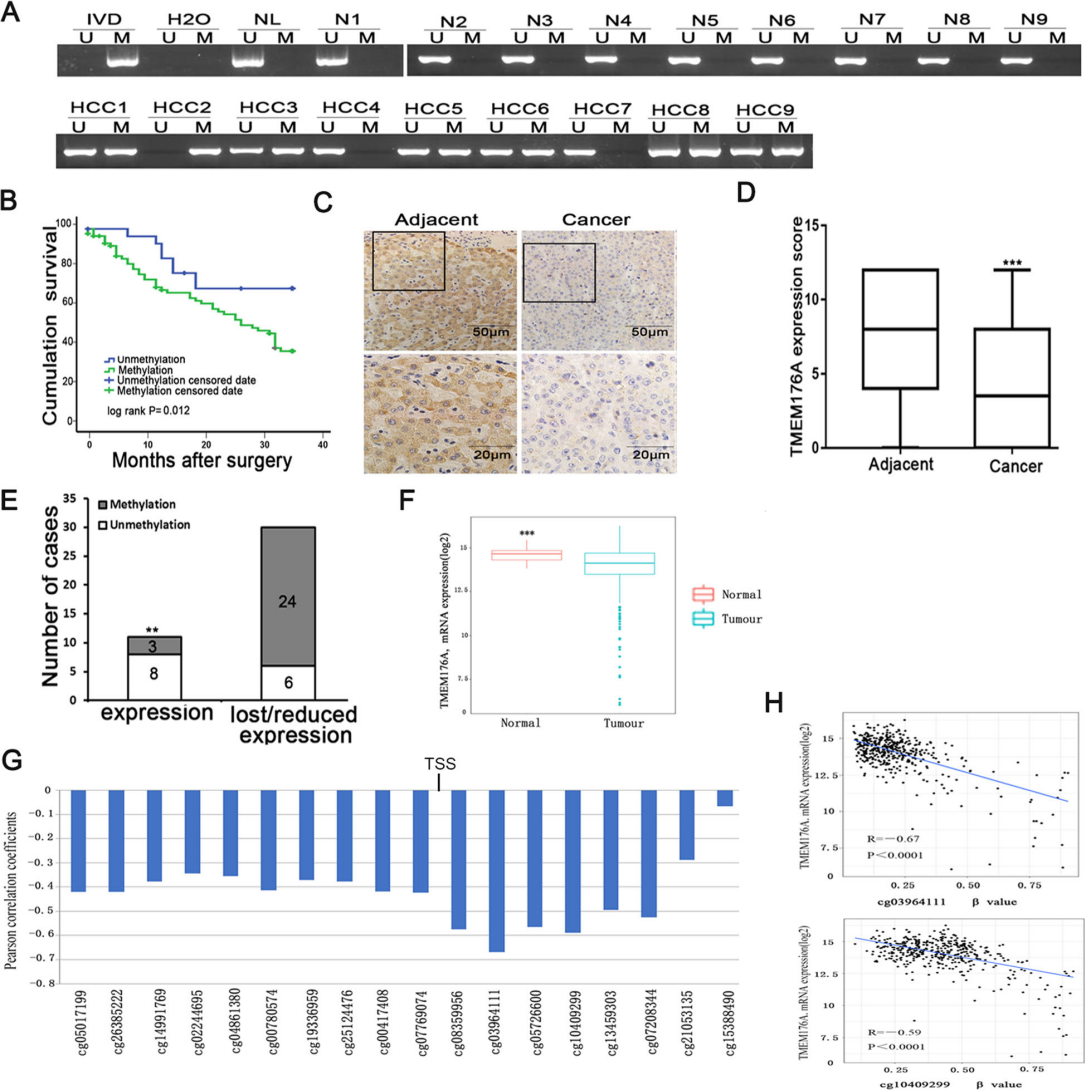
Expression and methylation status of TMEM176A in primary HCC. **a** Representative MSP results of TMEM176A in normal liver tissue samples and primary HCC samples. N: normal liver tissue samples; HCC: primary HCC samples. **b** The 3-year overall survival curves for patients in the methylated and unmethylated groups (*P* < 0.05). **c** Representative IHC results show TMEM176A expression in HCC tissue and adjacent tissue samples (top, × 200; bottom, × 400). **d** The expression of TMEM176A and DNA methylation status is shown as a bar diagram. Reduced expression of TMEM176A was significantly associated with promoter region hypermethylation. **P < 0.01. **e** TMEM176A expression scores are shown as box plots; horizontal lines represent the median score; the bottom and top of the boxes represent the 25th and 75th percentiles, respectively; vertical bars represent the range of data. Expression of TMEM176A was significantly different between adjacent tissue and HCC tissue in 41 matched HCC samples. ***P < 0.001. **f** TCGA data show TMEM176A mRNA expression levels in HCC tissues (*n* = 373) and normal liver tissues (*n* = 50) according to RNA-Seq results. Box plots, levels of TMEM176A expression. Horizontal lines, counts of log2 (TPM + 1). TPM: transcripts per million (reads). ***P < 0.001. **g** Pearson correlation coefficient between TMEM176A methylation and expression at each CpG site. **h** Scatter plots showing the methylation status of the 12th (cg03964111) and 14th (cg10409299) CpG sites, which are correlated with loss or reduced TMEM176A expression in 373 cases of HCC tissue samples. ***P < 0.001

The methylation status of TMEM176A was detected by MSP in 126 cases of human HCC and 15 cases of non-cancerous liver tissue samples. TMEM176A was methylated in 75.4% (90/126) of human primary HCC, and no methylation was found in non-cancerous liver tissue samples (Fig. 2a). Methylation of TMEM176A was significantly associated with tumor cell differentiation (*P* < 0.05, Table 1), while no association was found between TMEM176A methylation and age, gender, HBV infection, liver cirrhosis, tumor size, lymph node metastasis, vessel cancerous embolus, number of lesions, and TNM stage. According to Kaplan-Meier analysis, TMEM176A methylation was associated with poor 3-year OS (hazard ratio = 2.388, *P <* 0.05). Using multivariate analysis, TMEM176A methylation was associated with poor 3-year OS (hazard ratio = 2.370, *P* < 0.05, Fig. 2b, Table 2), suggesting that TMEM176A methylation is an independent prognostic factor for poor 3-year OS.

**Table 1:** The association of TMEM176A methylation and clinical factors in human HCC

Clinical factor	No.	TMEM176Amethylation status		*P* value
		Unmethylated, *n* = 31 (24.6%)	Methylated, *n* = 95 (75.4%)	
Age (years)				0.769
< 60	88	21	67	
≥ 60	38	10	28	
Gender				0.798
Male	108	27	81	
Female	18	4	14	
HBV infection				0.948
Yes	90	22	68	
No	36	9	27	
Liver cirrhosis				0.955
Yes	93	23	70	
No	33	8	25	
Tumor size (cm)				0.242
> 5	84	18	66	
≤ 5	42	13	29	
Differentiation				0.029*
Well or moderate	68	22	46	
Poor	58	9	49	
TNM stage				0.377
Stage I + stage II	52	14	38	
Stage III + stage IV	65	13	52	
Number of lesions				0.347
1	98	26	72	
≥ 1	28	5	23	
Vessel cancerous embolus				0.599
Negative	93	24	69	
Positive	33	7	26	
Lymph node metastasis				0.644
Negative	120	30	90	
Positive	6	1	5	

*P* values are obtained from chi-square test, significant difference

*P < 0.05

**Table 2:** Univariate and multivariate analysis of TMEM176A methylation status with 3 year-overall survival (OS) in HCC patients

Clinical factor	3-year OS			
	Univariate analysis		Multivariate analysis	
	HR (95% CI)	*P* value	HR (95% CI)	*P* value
Age	1.204	0.557		
(< 60 vs. ≥ 60 years)	(0.649–2.235)			
Gender	0.869	0.775		
(Male vs. female)	(0.331–2.282)			
TMEM176A	2.388	0.027*	2.370	0.025*
(Methylation vs. unmethylation)	(1.102–5.175)		(1.116–5.034)	
HBV infection	0.592	0.142		
(YES vs. NO)	(0.294–1.191)			
Liver cirrhosis	1.360	0.401		
(Yes vs. no)	(0.664–2.784)			
Tumor size	1.184	0.670		
(≤ 5 cm vs. > 5 cm)	(0.544–2.580)			
Number of lesions	0.902	0.793		
(1 vs. ≥ 1)	(0.419–1.944)			
Differentiation	2.212	0.020*	1.989	0.015*
(Well or moderate vs. poor)	(1.133–4.320)		(1.140–3.470)	
TNM stage	1.150	0.724		
(Stage I + stage II vs. stage III + stage IV)	(0.528–2.505)			
Lymph node metastasis	1.184	0.670		
(Negative vs. positive)	(0.544–2.580)			
Vessel cancerous embolus	0.444	0.025*	0.441	0.006**
(Negative vs. positive)	(0.219–0.902)		(0.245–0.794)	

*HR* hazard ratio

*P < 0.05; **P < 0.01

The expression of TMEM176A was evaluated by immunohistochemistry in 41 cases of available matched HCC and adjacent tissue samples. TMEM176A staining was found mainly in the cytoplasm and cell membranes (Fig. 2c). Lower-level expression of TMEM176A was found in 30 cases. The expression levels of TMEM176A were reduced in cancer compared to adjacent tissue samples (Fig. 2d, Student’s *t* distribution (*t* test), *P* < 0.05). Among the 30 cases that had reduced expression of TMEM176A, 24 cases were methylated. The reduced expression of TMEM176A was significantly associated with promoter region methylation (Fig. 2e, *t* test, *P* < 0.05). These data indicate that the expression of TMEM176A is regulated by promoter region methylation in human primary HCC.

The Cancer Genome Atlas (TCGA) database was employed to further validate that the expression of TMEM176A is regulated by promoter region methylation. TMEM176A mRNA expression and promoter region methylation data were extracted from TCGA database (http://xena.ucsc.edu/). Methylation of TMEM176A was analyzed by Illumina Infinium Human Methylation 450 (HM450). TMEM176A expression data were obtained by RNA sequencing from 373 cases of HCC and 50 cases of normal liver tissue samples. The expression level of TMEM176A was significantly decreased in HCC compared to normal liver tissue (*t* test, *P* < 0.001, Fig. 2f). In the 373 cases of HCC samples, reduced expression of TMEM176A was associated with promoter region hypermethylation (Fig. 2g, h). These data further suggested that the expression of TMEM176A is regulated by promoter region methylation.

### TMEM176A inhibits HCC cell proliferation

**Fig. 3:**
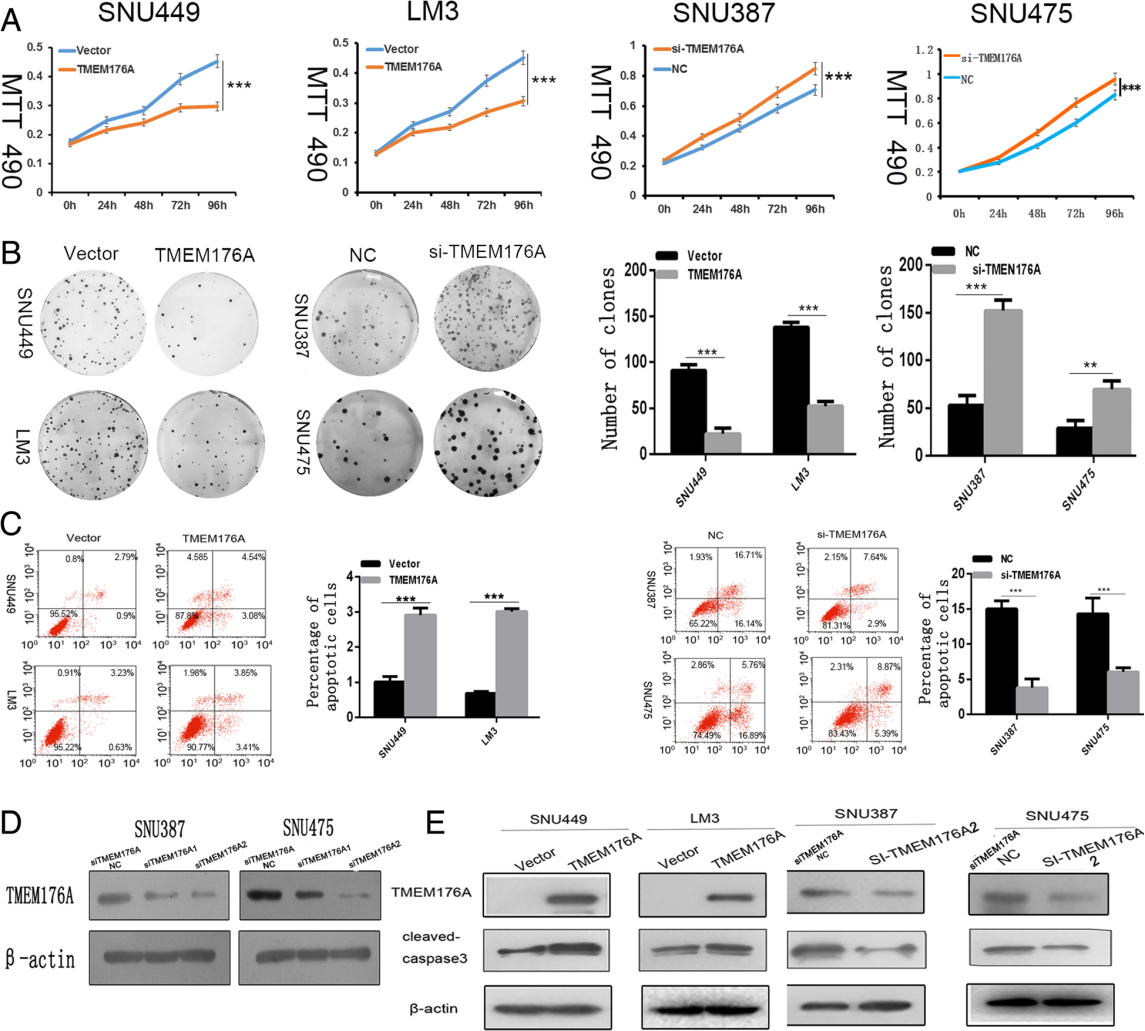
Effect of TMEM176A on HCC cell proliferation and apoptosis. **a** Growth curves represent cell viability analyzed by the MTT assay in TMEM176A re-expressed and unexpressed LM3 and SNU449 cells, as well as in SNU387 and SNU475 cells before and after knockdown of TMEM176A. Each experiment was repeated in triplicate. *P < 0.05, ***P < 0.001. **b** Colony formation results show that colony numbers were reduced by re-expression of TMEM176A in LM3 and SNU449 cells, while they were increased by knockdown of TMEM176A in SNU387 and SNU475 cells. Each experiment was repeated in triplicate. Average number of tumor clones is represented by bar diagram. *P < 0.05, ***P < 0.001. **c** Flow cytometry results show induction of apoptosis by re-expression of TMEM176A in LM3 and SNU449 cells, while reduction of apoptosis was found after knockdown of TMEM176A in SNU387 and SNU475 cells. *P < 0.05,***P < 0.001. **d** Knockdown of TMEM176A in SNU387 and SNU475 cells by siRNA. TMEM176A expression was examined by Western blots. SiTMEM176ANC: SiRNA for TMEM176A negative control; SiTMEM176A1: SiRNA for TMEM176A set1; SiTMEM176A2: SiRNA for TMEM176A set2. **e** Western blots show the effects of TMEM176A on the levels of cleaved caspase-3 expression in LM3, SNU449, SNU387, and SNU475 cells. VECTOR: control vector, TMEM176A: TMEM176A expressing vector, β-actin: internal control, NC: siRNA negative control

MTT and colony formation assays were used to evaluate the effects of TMEM176A on cell proliferation. TMEM176A stably expressed cells were established by transfection assay, and TMEM176A highly expressed cells were knocked down by siRNA. The OD values were 0.451 ± 0.023 vs. 0.3065 ± 0.017 in LM3 cells (*t* test, *P* < 0.05) and 0.452 ± 0.012 vs. 0.300 ± 0.019 (*t* test, *P* < 0.05) in SNU449 cells before and after the restoration of TMEM176A expression (Fig. 3a). The OD values were reduced significantly after the restoration of TMEM176A expression in LM3 and SNU449 cells (*t* test, both *P* < 0.001). The OD values were 0.833 ± 0.025 vs. 0.96 ± 0.040 (*t* test, *P* < 0.05) in SNU475 and 0.709 ± 0.021 vs. 0.848 ± 0.019 (*t* test, *P* < 0.05) in SNU387 before and after the knockdown of TMEM176A (Fig. 3a). The OD values increased significantly after the knockdown of TMEM176A expression in SNU387 and SNU475 cells (*t* test, both *P* < 0.001). These results demonstrated that TMEM176A inhibits cell proliferation in HCC cells. The clone numbers were 138 ± 5.2 vs. 52.3 ± 4.9 in LM3 cells (*t* test, *P* < 0.05) and 90.7 ± 6.3 vs. 22.3 ± 6.1 in SNU449 cells (*t* test, *P* < 0.05) before and after the restoration of TMEM176A expression (Fig. 3b). The clone numbers were 29 ± 7.9 vs. 79.7 ± 9 (*t* test, *P* < 0.01) in SNU475 cells and 53 ± 10.4 vs. 152 ± 11.4 (*t* test, *P* < 0.01) in SNU387 cells before and after the knockdown of TMEM176A (Fig. 3b). These data suggest that TMEM176A suppresses cell growth in HCC.

### TMEM176A induces HCC cell apoptosis

The effect of TMEM176A on apoptosis was analyzed by flow cytometry. Under doxorubicin treatment, the ratios of apoptotic cells in TMEM176A unexpressed and re-expressed cells were 0.69 ± 0.08% vs. 3.01 ± 0.05% in LM3 cells and 1.01 ± 0.19% vs. 2.91 ± 0.15% in SNU449 cells. The ratio of apoptotic cells increased significantly after the re-expression of TMEM176A (*t* test, *P* < 0.05 for both cells; Fig. 3c). In SNU387 and SNU475 cells, the ratios of apoptotic cells were 15.00 ± 1.16% vs. 3.81 ± 0.86% and 14.3 ± 2.24% vs. 6.06 ± 0.58%, respectively, before and after the knockdown of TMEM176A. The ratio of apoptotic cells decreased significantly after the knockdown of TMEM176A (*t* test, *P* < 0.05, Fig. 3c). To further validate the effect of TMEM176A on apoptosis, cleaved caspase-3 expression was analyzed in HCC cells. The levels of cleaved caspase-3 increased after the re-expression of TMEM176A in LM3 and SNU449 cells and decreased after the knockdown of TMEM176A in SNU475 and SNU387cells (Fig. 3d). These results demonstrate that TMEM176A induces apoptosis in HCC cells (Fig. 3e).

### TMEM176A inhibits HCC cell migration and invasion

**Fig. 4:**
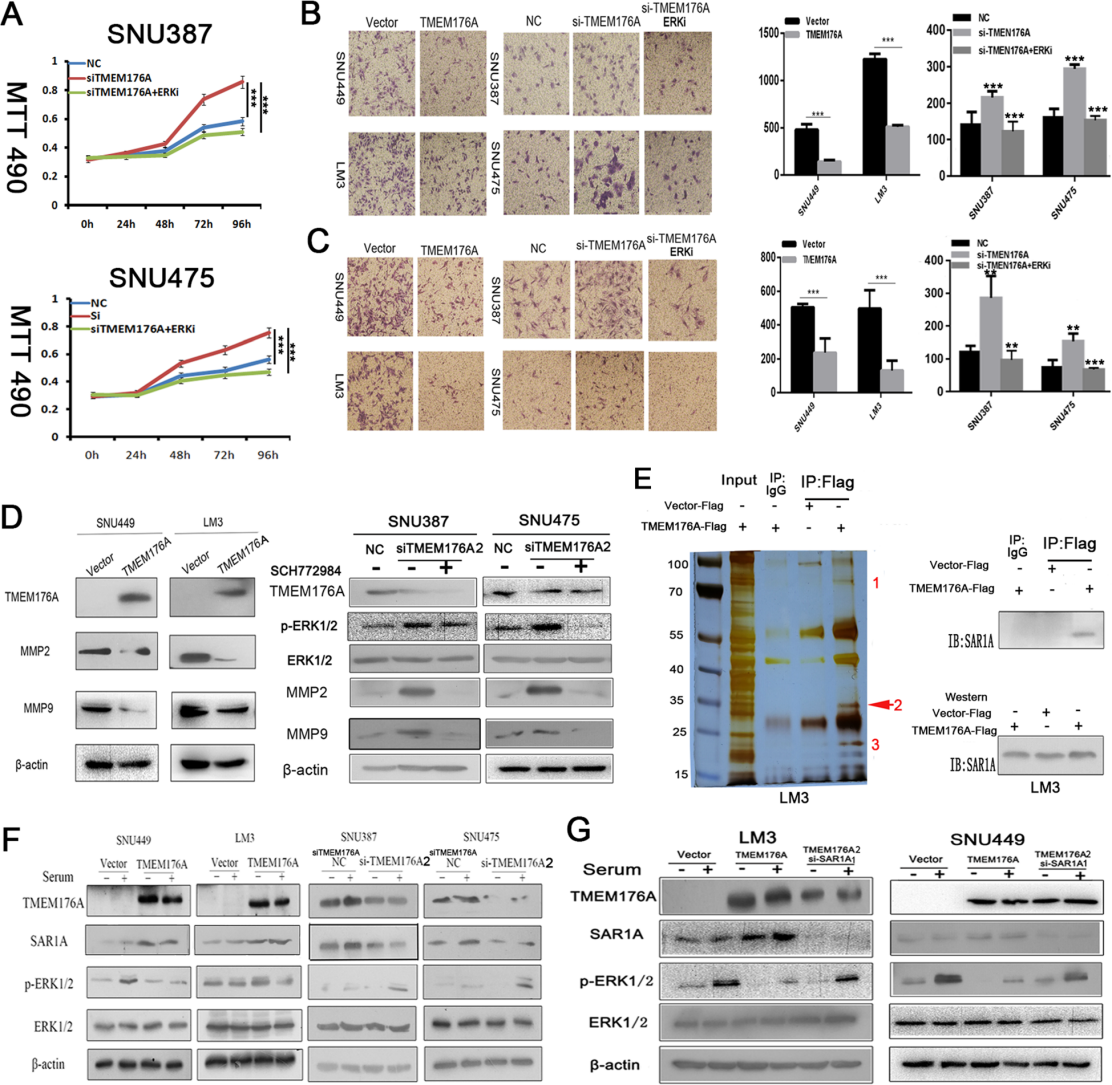
TMEM176A inhibits HCC cells invasion, migration, and the ERK signaling pathway. **a** Growth curves represent cell viability evaluated by MTT assay in the control group, siTMEM176A group, and siTMEM176A plus SCH772984 treatment group. siTMEM176A: siRNA knockdown of TMEM176A. ***P < 0.001. **b** The migration assays show migration cells before and after restoration of TMEM176A expression in SNU449 and LM3 cells as well as in the control group, siTMEM176A group, and siTMEM176A plus SCH772984 treatment group. The number of cells migrating to the lower chamber is presented by bar diagram. Each experiment was repeated for three times. **P < 0.01, ***P < 0.001. **c** The invasion assays show invasive cells before and after restoration of TMEM176A expression in SNU449 and LM3 cells as well as in the control group, siTMEM176A knockdown group, and siTMEM176A plus SCH772984 treatment group. The invasion cell number is presented by bar diagram. Each experiment was repeated three times. **P < 0.01, ***P < 0.001. **d** The expression levels of TMEM176A, MMP2, and MMP9 were detected by Western blot. **e** LM3 cells were transfected with pcDNA3.1-TMEM176A-Flag or Vector-Flag. Immunoprecipitation was performed using an anti-Flag antibody or Rabbit IgG antibody. The bands specific to TMEM176A, as pointed out by the numbers (bands 1–3), were subjected to mass spectrometry. The TMEM176A (band 2) is indicated by the red arrow. LM3 cells were transfected with pcDNA3.1-TMEM176A-Flag or Vector-Flag. Immunoblots showing SAR1A in the anti-Flag-TMEM176A immunoprecipitates. Input: cell lysis of TMEM176A re-expressed LM3 cells; IgG: negative control. **f** Western blots show the levels of TMEM176A, SAR1A, ERK1/2, and p-ERK1/2 in SNU449, LM3, SNU387, and SNU475 cells. β-actin: internal control. -: no serum stimulation. +: serum stimulation. **g** Western blots show the levels of TMEM176A, SAR1A, ERK1/2, and p-ERK1/2 in the control group and siSAR1A knockdown group. SiSAR1ANC: SiRNA for SAR1A negative control; SiSAR1A1: SiRNA for SAR1A set1. β-actin: internal control. -: no serum stimulation. +: serum stimulation

To evaluate the effects of TMEM176A on cell migration and invasion, transwell assays were used. The numbers of migration cells were 1233.6 ± 61.3 vs. 508.8 ± 18.1 in LM3 cells and 479.75 ± 58.80 vs. 143.00 ± 15.20 in SNU449 cells before and after the restoration of TMEM176A expression. The number of migration cells decreased significantly after the re-expression of TMEM176A in LM3 and SNU449 cells (*t* test, both *P* < 0.001, Fig. 4b). The numbers of migration cells were 162.00 ± 21.8 vs. 299.44 ± 22.28 in SNU475 cells and 140.28 ± 35.86 vs. 215.86 ± 17.16 in SNU387 cells before and after the knockdown of TMEM176A. The number of migration cells increased significantly after the knockdown of TMEM176A in SNU475 and SNU387 cells (*t* test, *P* < 0.001, Fig. 4b). The numbers of invasion cells were 496.4 ± 60.48 vs. 131.2 ± 29.9 in LM3 cells and 489.33 ± 79.48 vs. 250.33 ± 42.25 in SNU449 cells before and after the restoration of TMEM176A expression. The cell number decreased significantly after the re-expression of TMEM176A in LM3 and SNU449 cells (*t* test, both *P* < 0.001, Fig. 4b). The numbers of invasion cells were 74.5 ± 21.93 vs. 153.25 ± 23.04 in SNU475 cells and 100.47 ± 19.32 vs. 242.14 ± 66.69 in SNU387 cells before and after the knockdown of TMEM176A. The cell number increased significantly after knockdown of TMEM176A in SNU475 and SNU387 cells (*t* test, *P* < 0.01, Fig. 4c). These results suggest that TMEM176A suppresses HCC cell migration and invasion. To further explore the mechanism of TMEM176A on cell migration and invasion, MMP2 and MMP9 expression were measured by Western blot. The expression levels of MMP2 and MMP9 were reduced after the re-expression of TMEM176A in LM3 and SNU449 cells. However, the expression levels of MMP2 and MMP9 increased after the knockdown of TMEM176A in SNU475 and SNU387 cells (Fig. 4d). These results suggest that TMEM176A inhibits cell invasion in HCC cells. According to our above study in four cell lines, silencing of TMEM176A expression by promoter region methylation promotes cell migration, invasion, or metastasis.

### TMEM176A inhibits ERK signaling pathway in HCC cells

To further explore the molecular mechanism of TMEM176A in HCC, immunoprecipitation assays were performed using anti-Flag antibody in TMEM176A unexpressed and re-expressed LM3 cells. TMEM176A unexpressed and re-expressed cellular proteins were captured by Protein A agarose beads and then subjected to sodium dodecyl sulphate-polyacrylamide gel electrophoresis (SDS-PAGE) analysis (Fig. 4e). Three protein bands were found to be specifically associated with TMEM176A by comparison to the protein bands from TMEM176A re-expressed LM3 cells and unexpressed LM3 cells. The protein bands specifically associated with TMEM176A were then extracted, digested with trypsin, and subjected to mass spectrometry analysis. TMEM176 binding proteins were selected among those associated with cancer-related signaling pathways to validate by immunoprecipitation assay. Secretion-associated Ras-related GTPase 1A (SAR1A) was clearly detected in TMEM176A-Flag pull-down proteins; however, it was not detected in the complexes associated with Flag or IgG groups (Fig. 4e). The results were validated by Western blot in LM3 cells (Fig. 4f). The interaction of SAR1A and TMEM176A was further validated by Western blot in SNU449, SNU387, and SNU475 cells. As shown in Fig. 4f, the SAR1A protein band density increased after the re-expression of TMEM176A in SNU449 cells. The density of the SAR1A protein band was reduced after the knockdown of TMEM176A in TMEM176A highly expressed SNU387 and SNU475 cells.

SAR1A belongs to the SAR superfamily and encodes a GTP-binding protein SAR1A. SAR1A was reported to inhibit the ERK signaling pathway in K562 cells [25]. To further understand the mechanism of TMEM176A in HCC, the role of TMEM176A in ERK signaling was investigated. The levels of total ERK1/2 and phosphorylated ERK1/2 (p-ERK1/2) were detected by Western blot in HCC cells with or without TMEM176A expression. As shown in Fig. 4f, no apparent difference was found for the levels of ERK1/2 before and after the re-expression of TMEM176A in LM3 and SNU449 cells. The levels of p-ERK1/2 were reduced after the re-expression of TMEM176A in LM3 and SNU449 cells. The levels of ERK1/2 were similar in TMEM176A highly expressed and siRNA knockdown in SNU387 and SNU475 cells. The levels of p-ERK1/2 increased after the knockdown of TMEM176A in SNU387 and SNU475 cells. These results suggest that TMEM176A inhibits ERK signaling in HCC.

To further validate our finding, ERK1/2 inhibitor (SCH772984) was employed. The OD values were 0.583 ± 0.086, 0.857 ± 0.032, and 0.510 ± 0.021 in the control group, siTMEM176A group, and siTMEM176A plus SCH772984 treatment group in SNU387 cells, respectively. The OD values were 0.546 ± 0.025, 0.754 ± 0.015, and 0.491 ± 0.031 in the control group, siTMEM176A group, and siTMEM176A plus SCH772984 treatment group in SNU475 cells, respectively. No significant difference was found between the control group and siTMEM176A plus SCH772984 treatment group (both *P* > 0.05) in SNU387 and SNU475 cells, while the OD value is reduced significantly in siTMEM176A plus SCH772984 treatment group compared to siTMEM176A group (both *P* < 0.001 Fig. 4a) in SNU387 and SNU475 cells. The above results further validated that TMEM176A inhibits ERK signaling in HCC.

The numbers of migrated cells for each microscopic field were 140.28 ± 35.86, 215.86 ± 17.16, and 122.86 ± 24.39 in the control group, siTMEM176A group, and siTMEM176A plus SCH772984 treatment group in SNU387 cells, respectively. The numbers of migrated cells for each microscopic field were 162.00 ± 21.8, 299.44 ± 22.28, and 152.86 ± 11.6 in the control group, siTMEM176A group, and siTMEM176A plus SCH772984 treatment group in SNU475 cells, respectively. No significant difference was found between the control group and siTMEM176A plus SCH772984 treatment group (both *P* > 0.05) in SNU387 and SNU475 cells. The numbers of migrated cells were reduced significantly in the siTMEM176A plus SCH772984 treatment group compared to the siTMEM176A group (*P* < 0.001, Fig. 4b). The numbers of invasion cells for each microscopic field were 100.47 ± 19.32, 242.14 ± 66.69, and 96.75 ± 28.01 in the control group, siTMEM176A group, and siTMEM176A plus SCH772984 treatment group in SNU387 cells, respectively. The numbers of invasion cells for each microscopic field were 74.5 ± 21.93, 153.25 ± 23.04, and 67.75 ± 4.86 in the control group, siTMEM176A group, and siTMEM176A plus SCH772984 treatment group in SNU475 cells, respectively. No significant difference was found between the control group and siTMEM176A plus SCH772984 treatment group (both *P* > 0.05) in SNU387 and SNU475 cells. The numbers of invasion cells were reduced significantly after treatment with SCH772984 in siTMEM176A group compared to siTMEM176A group (*P* < 0.01, *P* < 0.001, Fig. 4b).

To further verify that TMEM176A inhibit ERK pathway through SAR1A, the levels of p-ERK1/2 were detected in empty vector, TMEM176A stable expression, and TMEM176A stable expression plus siRNA knockdown SAR1A groups (siSAR1A) in LM3 and SNU449 cells. As shown in Fig. 4g, no difference was found for ERK1/2 levels in vector, TMEM176A stably expressed, and TMEM176A stable expression plus si-SAR1A LM3 and SNU449 cells. The levels of p-ERK1/2 were reduced after the re-expression of TMEM176A in LM3 and SNU449 cells. However, the levels of p-ERK1/2 were increased after the knockdown of SAR1A in TMEM176A stable expressed LM3 and SNU449 cells (Fig. 4g). These results further validated that TMEM176A inhibits ERK pathway by interacting with SAR1A.

### TMEM176A suppresses human HCC cell xenograft growth in mice

**Fig. 5:**
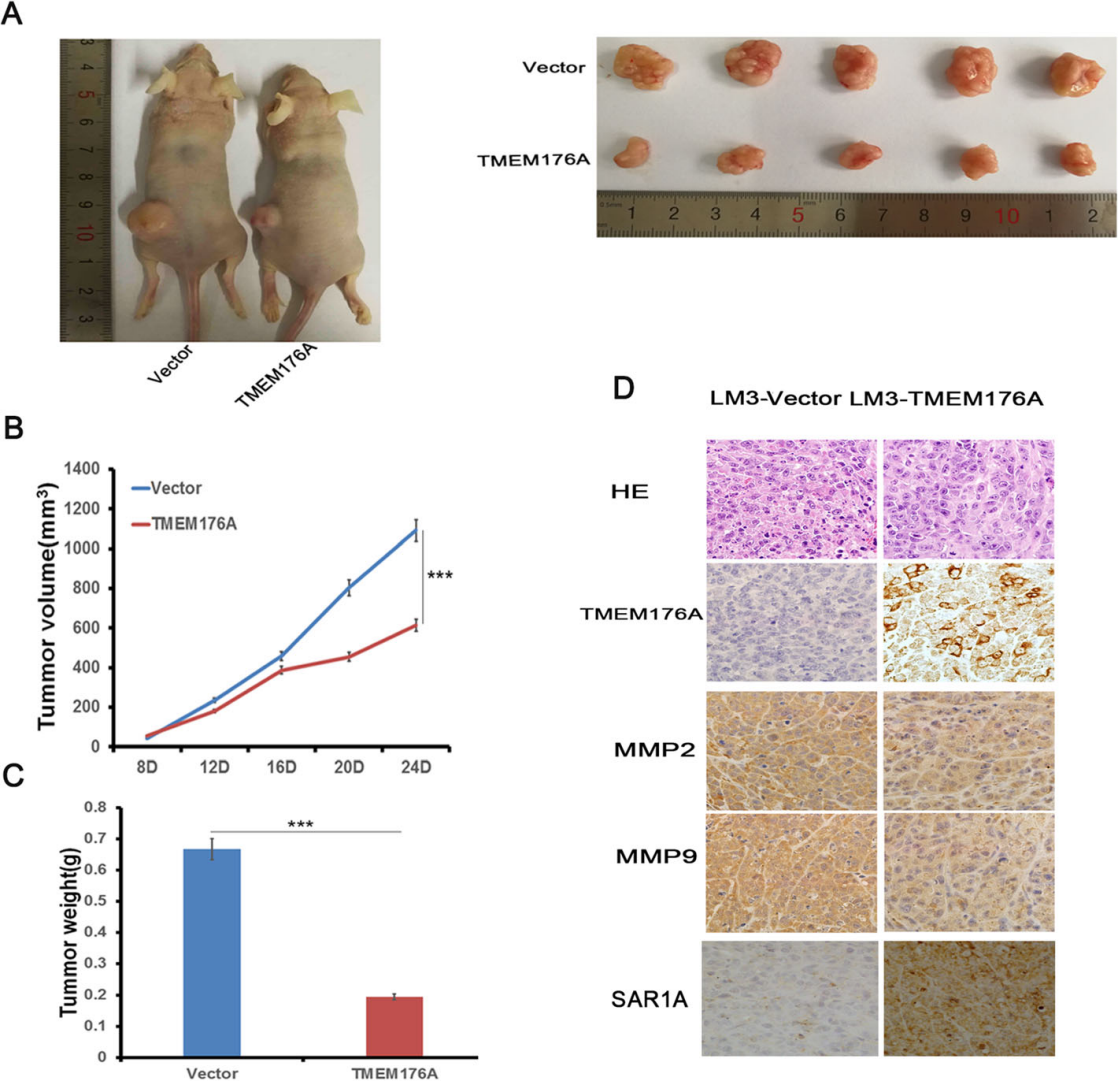
TMEM176A suppresses human HCC cell xenograft growth in mice. **a** Representative tumors from TMEM176A unexpressed and TMEM176A re-expressed LM3 cell xenografts. **b** Tumor growth curves of TMEM176A unexpressed and TMEM176A re-expressed LM3 cells. ***P < 0.001. **c** Tumor weights in nude mice at the 24th day after inoculation of unexpressed and TMEM176A re-expressed LM3 cells. Bars: mean of five mice. ***P < 0.001. **d** Images of hematoxylin and eosin staining show tumors from TMEM176A unexpressed and TMEM176A re-expressed LM3 xenograft mice. IHC staining reveals the expression levels of TMEM176A, MMP2, MMP9, and SAR1A in TMEM176A unexpressed and TMEM176A re-expressed LM3 cell xenografts. Clinical specimens of low and high expression of TMEM176A were stained for SAR1A (× 400)

To further evaluate the effect of TMEM176A in human HCC, TMEM176A unexpressed and re-expressed LM3 cells were used to establish the xenograft mouse models (Fig. 5a). The tumor volume was 1090.58 ± 62.48 vs. 614.43 ± 52.7 mm^3^ in TMEM176A unexpressed and re-expressed LM3 cell xenografts, respectively (Fig. 5b). The tumor volume was reduced significantly in TMEM176A re-expressed LM3 cell xenograft mice (*t* test, *P* < 0.001). The tumor weight was 0.67 ± 0.12 g vs. 0.19 ± 0.04 g in TMEM176A unexpressed and re-expressed LM3 cell xenograft mice, respectively (Fig. 5c). The tumor weight was reduced significantly in TMEM176A re-expressed LM3 cells xenograft mice (*t* test, *P* < 0.001). The results indicate that TMEM176A suppresses HCC cell growth in vivo. To further validate the effect of TMEM176A on tumor metastasis, the expression of MMP2 and MMP9 were examined by IHC in xenograft tumors. The expression levels of MMP2 and MMP9 were decreased in TMEM176A re-expressed LM3 cell xenografts compared to TMEM176A unexpressed LM3 cells (Fig. 5d). In addition, the expression of TMEM176A and SAR1A was found correlated very well in LM3 cell xenografts (Fig. 5d).

## Discussion

TMEM176A was reported to participate in the maintenance of the immature state of mouse dendritic cells [11, 26]. Most previous studies were mainly focused on the development and the immune system [15, 26–28]. In mouse, the loss of TMEM176B is associated with the upregulation of TMEM176A [29]. TMEM176A and B exhibit a similar cation channel activity and mainly co-localize in close proximity to the trans-Golgi network [29]. In our previous study, TMEM176A was found to be frequently methylated in human colorectal and esophageal cancers. In this study, we analyzed the function of TMEM176A in HCC both in vitro and in vivo and further explored the mechanism of TMEM176A in HCC. By analyzing the expression and promoter region methylation status in HCC cells, we found that loss of/reduced expression of TMEM176A is correlated with promoter region methylation. Re-expression of TMEM176A was induced by DAC in methylated HCC cells. These results suggest that the expression of TMEM176A is regulated by promoter region methylation. In primary HCC, we found that the loss of/reduced expression of TMEM176A is associated with promoter region methylation, indicating that the expression of TMEM176A may be regulated by promoter region methylation in primary HCC. To further validate our findings, data from the TCGA database were analyzed. This analysis indicated that the expression level of TMEM176A was significantly decreased in HCC, and reduced expression of TMEM176A was associated with promoter region hypermethylation. These results further suggested that the expression of TMEM176A is regulated by promoter region methylation in HCC. In our study, we performed methylation detection in 126 cases of HCC. We demonstrated that TMEM176A is frequently methylated in HCC. In addition, methylation of TMEM176A was associated with tumor differentiation and was an independent prognostic factor for poor 3-year OS. These results suggest that TMEM176A methylation may serve as a diagnostic and poor prognostic marker in HCC.

To rule out the bias of different levels of TMEM176A expression in cell models, we selected two cell lines (LM3 and SNU449), which were completely methylated and unexpressed, for re-expression study. We also selected SNU387 and SNU475 cells, which were unmethylated and highly expressed, for siRNA knockdown experiment. Next, we analyzed the function of TMEM176A both in HCC cells and in vivo.

TMEM176A induced cell apoptosis and inhibited cell proliferation, migration, and invasion in HCC. TMEM176A suppressed tumor growth in xenograft mice. Thus, TMEM176A may serve as a tumor suppressor in human HCC.

To further explore the molecular mechanism of TMEM176A in HCC, immunoprecipitation assays and mass spectrometry analysis were performed. SAR1A protein was identified to bind TMEM176A in human HCC cells. The small GTP-binding protein superfamily comprises more than 100 members in eukaryotes [30]. SAR itself functions in cargo selection and export of proteins from the endoplasmic reticulum to the Golgi via the cytosolic coat protein complex II (COPII) secretory pathway. Tang et al. demonstrated that SAR participates in both erythroid cell growth and γ-globin production by regulating the ERK signaling pathway [25]. Our further analysis demonstrated that TMEM176A inhibited the ERK signaling pathway by interacting with SAR1A in human HCC both in vitro and in vivo.

## Conclusion

In conclusion, TMEM176A is frequently methylated in human HCC, and the expression of TMEM176A is regulated by promoter region methylation. Methylation of TMEM176A may serve as a diagnostic and prognostic marker in HCC. TMEM176A suppresses HCC growth by inhibiting the ERK signaling pathway.

## Abbreviations

BSSQ: Bisulfite sequencing
DAC: 5-Aza-2′-deoxycytidine
GAPDH: Glyceraldehyde-3-phosphate dehydrogenase
HCC: Hepatocellular carcinoma
HM450: Illumina Infinium Human Methylation 450
IHC: Immunohistochemistry
IVD: In vitro-methylated DNA
MSP: Methylation-specific PCR
NL: Normal lymphocyte DNA
OS: Overall survival
RT-PCR: Reverse transcription PCR
SAR1A: Secretion associated Ras-related GTPase 1A
TCGA: The Cancer Genome Atlas
TSS: Transcription start sites
